# Comparison of net ecosystem carbon exchange estimation in a mixed temperate forest using field eddy covariance and MODIS data

**DOI:** 10.1186/s40064-016-2134-4

**Published:** 2016-04-21

**Authors:** Yuandong Wang, Xuguang Tang, Lianfang Yu, Xiyong Hou, J. William Munger

**Affiliations:** Key Laboratory of Digital Earth Science, Institute of Remote Sensing and Digital Earth, Chinese Academy of Sciences, Beijing, 100094 China; College of Geography and Planning, Gannan Normal University, Ganzhou, 341000 China; Nanjing Institute of Geography and Limnology, Chinese Academy of Sciences, Nanjing, 210008 China; Yantai Institute of Coastal Zone Research, Chinese Academy of Sciences, Yantai, 264003 China; School of Engineering and Applied Sciences, Harvard University, Cambridge, MA USA

**Keywords:** NEE, Eddy covariance, MODIS, PAR, EVI, LST

## Abstract

Quantification of net ecosystem carbon exchange (NEE) between the atmosphere and vegetation is of great importance for regional and global studies of carbon balance. The eddy covariance technique can quantify carbon budgets and the effects of environmental controls for many forest types across the continent but it only provides integrated CO_2_ flux measurements within tower footprints and need to be scaled up to large areas in combination with remote sensing observations. In this study we compare a multiple-linear regression (MR) model which relates enhanced vegetation index and land surface temperature derived from the moderate resolution imaging spectroradiometer (MODIS), and photosynthetically active radiation with the site-level NEE, for estimating carbon flux exchange between the ecosystem and the environment at the deciduous-dominated Harvard Forest to three other methods proposed in the literature. Six years (2001–2006) of eddy covariance and MODIS data are used and results show that the MR model has the best performance for both training (2001–2004, *R*^2^ = 0.84, *RMSE* = 1.33 g Cm^−2^ day^−1^) and validation (2005–2006, *R*^2^ = 0.76, *RMSE* = 1.54 g Cm^−2^ day^−1^) datasets comparing to the other ones. It provides the potential to estimate carbon flux exchange across different ecosystems at various time intervals for scaling up plot-level NEE of CO_2_ to large spatial areas.

## Background

Quantification of the net carbon exchange between atmosphere and terrestrial ecosystem in global carbon cycle is becoming important with future potential sequestration influenced by increased atmospheric CO_2_ and changing climate (Nemani et al. 2003). Therefore, accurately estimating the net ecosystem carbon exchange (NEE), which is the difference between photosynthetic uptake and release of CO_2_ by respiration from autotrophs (vegetation) and heterotrophs (free living fauna in the soil and symbiotic microorganisms), at the regional, continental or global scale, is helpful to improve our understanding of the feedbacks between terrestrial biosphere and atmosphere in the context of global change and facilitate climate policy-making (Canadell et al. 2000; Xiao et al. 2010; Tang et al. 2011; Hu et al. 2014).

Traditionally, inventory studies of biomass and soil carbon were used to quantify an ecosystem NEE over a specific period (Clark et al. 2001). In recent years, the development of eddy covariance technique provides an alternative approach to continuously measure long term carbon exchange at ecosystem scales and evaluating carbon balance as well as its seasonal or annual variations more precisely has become possible (Baldocchi et al. 2001). Carbon budgets and the effects of environmental controls have been quantified with this technique for many forest types across the continent (Powell et al. 2006; Crawford and Christen 2014). However, the EC technique only provide integrated CO_2_ flux measurements over tower footprints with sizes and shape that vary with tower height, canopy physical characteristics and wind velocity (Osmond et al. 2004). Scaling up beyond the tower footprint to large areas is critically important in the quantification of net CO_2_ exchange over regions or continents (Gitelson et al. 2006, 2012; Xiao et al. 2010). Satellite remote sensing provides ecosystem observations with temporally and spatially coverage, and is an attractive and powerful tool for up-scaling carbon fluxes. A number of remote sensing based ecosystem carbon exchange models have been proposed recently to extend the role of field plots to capture regional variation and to bridge a major gap between field and satellite observations (Gregory et al. 2010). For example, Gamon et al. (1997) propose the photochemical reflectance index (PRI) that can correlate with light use efficiency (LUE) for carbon exchange estimation at leaf, canopy, stand and landscape levels (Gamon et al. 1997, 2001; Rahman et al. 2001, 2005). Vegetation indices (VI) such as NDVI and the enhanced vegetation index (EVI) are also used to directly estimate carbon fluxes (Xiao et al. 2004; Sims et al. 2006; Wu et al. 2010, 2012). Gitelson et al. (2006) first introduce the greenness and radiation (GR) model utilizing the total chlorophyll vegetation index and photosynthetically active radiation (PAR) to estimate carbon fixation in crops with high accuracy. The temperature and greenness (TG) model developed by Sims et al. (2008) that based on the MODIS EVI and land surface temperature (LST) product is validated in a wide diversity of natural vegetation including both deciduous and evergreen forests across North America. These studies demonstrate that greenness indices like enhanced vegetation indices (EVI) and land surface water index (LSWI), land surface temperature (LST), photosynthetically active radiation (PAR) are reliable proxies indicating plant phenological stages, canopy stresses (air temperature, soil moisture, vapor pressure deficit) and environmental conditions (incoming solar radiation) in estimation of carbon uptake by terrestrial ecosystems referred to as gross ecosystem exchange (GEE), but the ability of these biophysical indices in capturing the net carbon uptake by forest ecosystems namely NEE is less well known. Therefore, the objectives of this study are: (1) to analyze the potential of EVI, LSWI, LST, and PAR in tracking NEE seasonal dynamics, (2) on the basis of previous studies, to compare a newly proposed MR model with other models for NEE estimation in the Harvard deciduous broadleaf forest by selectively incorporating these proxies. This study will explore the implication and ability of eddy covariance and remote sensing observations for quantifying net carbon exchange between the atmosphere and forest ecosystems.

## Methods

### Study site

**Fig. 1:**
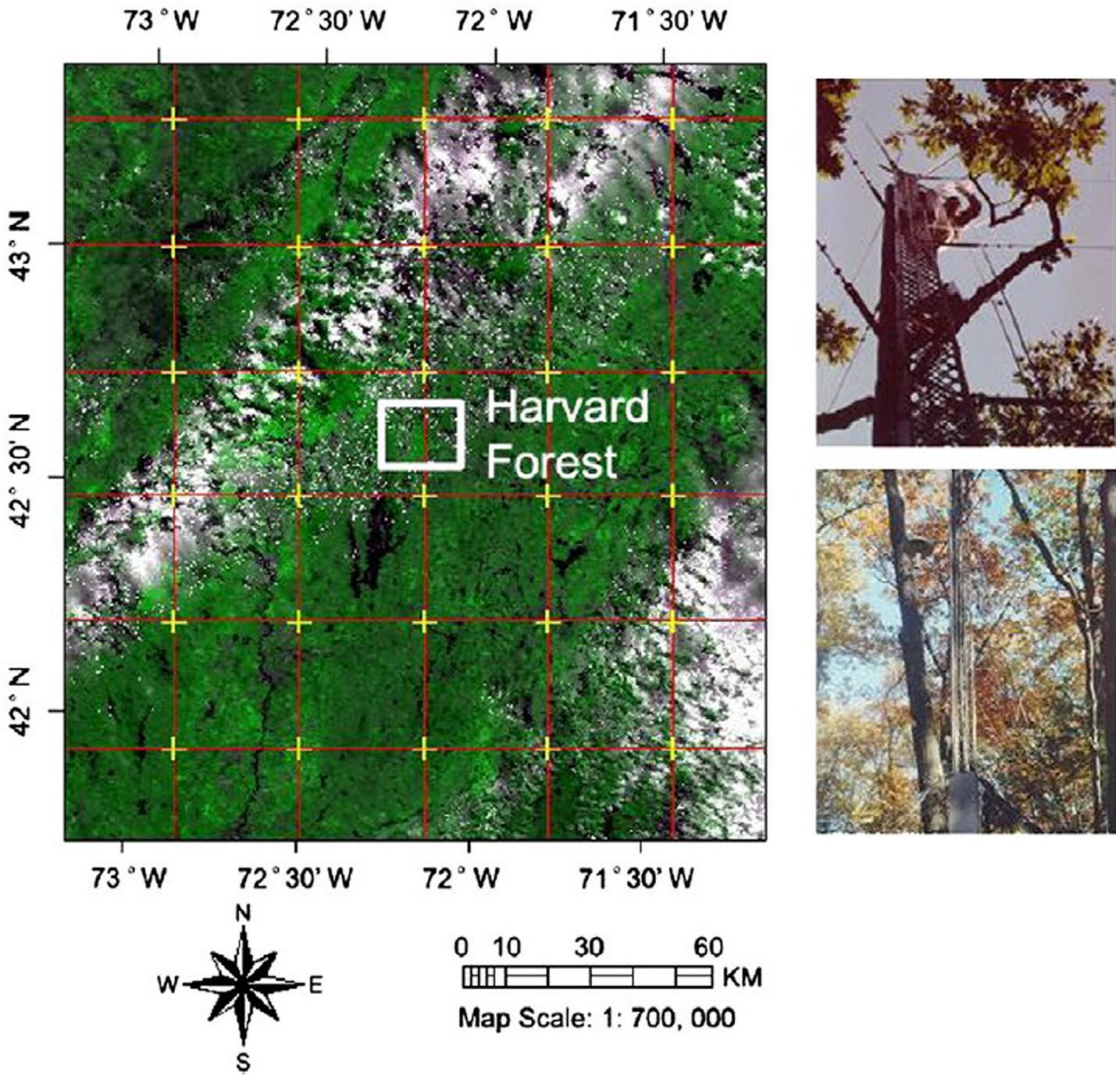
Study area in view using MODIS image of 2009 (DOY of 121), photos were downloaded from http://atmos.seas.harvard.edu/lab/hf/hfsite.html

The Harvard eddy flux tower (42°32′16″N and 72°10′17″W, 340 m elevation) is located in a mixed temperate forest, approximately 110 km west of Boston, Massachusetts, USA (Fig. 1). The vegetation is mainly deciduous forest, dominated by red oak (*Quercus rubra*), red maple (*Acer rubrum*), black birch (*Betula lenta*) and white pine (*Pinus strobus*). The canopy height is approximately 20–24 m. Soil composition are primarily sandy loam glacial till with some alluvial and colluvial deposits. The climate is cool, moist temperate with mean annual temperature 6.5 °C. Annual mean precipitation is 1000 mm and distributed approximately evenly throughout the year. Plant growing season usually start around mid-May (~day of year 130) and lasts about 160 days (Urbanski et al. 2007).

### Eddy covariance data

The direct measurement of long-term carbon fluxes by eddy covariance technique provides the possibility of estimating local carbon sequestration rates of forests and different land use types. EC also improves our understanding of the vulnerability of ecosystem carbon balance to climate changes. Furthermore, it can help to evaluate ecosystem models and provide data for land surface exchange schemes in global models (Valentini et al. 2000). Eddy flux measurements of CO_2_, H_2_O and energy at Harvard Forest have been collected since 1991. The 6-year measurements (2001–2006) of NEE of CO_2_, daily PAR data are provided by researchers at Harvard Forest (http://public.ornl.gov/ameriflux/). Site-specific procedures, including quality control, flux corrections, and data editing are described elsewhere (Barford et al. 2001; Urbanski et al. 2007). The level 4 product consists of NEE data with four different time steps, including half-hourly, daily, weekly (8-day) and monthly. We utilize the weekly NEE data and the sums of PAR calculated over 8-day periods to match the compositing intervals of MODIS. The average of NEE and PAR over such a period was shown to largely eliminate micrometeorological errors caused by variable weather conditions or sampling procedures, with the remaining variability representing variation in ecosystem attributes (Oren et al. 2006).

### MODIS data

The MODIS is a key instrument aboard the Terra and Aqua satellites, acquiring data in 36 spectral bands from 450 to 2100 nm. Two collection 5 MODIS products the 8-day land surface reflectance (MOD09A1) and land surface temperature (MOD11A2) from 2001 to 2006 are obtained from the 7 × 7 km subsets of MODIS products available at Oak Ridge National Laboratory’s Distributed Active Archive Center web site (http://www.modis.ornl.gov.modis.index.cfm). Average values of the central 3 × 3 km are extracted within the 7 × 7 km cutouts to better represent the flux tower footprint (Rahman et al. 2005).

Reflectance values of four spectral bands blue, red, near infrared (841–875 nm), and shortwave infrared (1628–1652 nm) in 2001–2006 are used to compute two vegetation greenness indices of the enhanced vegetation index (EVI) and the Land Surface Water Index (LSWI). EVI directly normalizes the reflectance in the red band as a function of the reflectance in blue band:

1 $${\text{EVI}} = 2.5 \times \frac{{{\text{R}}_{\text{Nir}} - {\text{R}}_{\text{Red}} }}{{1 + {\text{R}}_{\text{Nir}} + 6 \times {\text{R}}_{\text{Red}} - 7.5 \times {\text{R}}_{\text{Blue}} }}$$

EVI is designated to primarily account for residual atmospheric reflectance, variable soil and canopy background reflectance (Huete et al. 2002). It has been successfully used for in temperate forests and shown to be much less sensitive to aerosols than NDVI (Xiao et al. 2003). The LSWI proposed by Xiao et al. (2002) is derived from the combination of near infrared (NIR) and shortwave infrared (SWIR) bands using the following equation:

2 $${\text{LSWI}} = \frac{{{\text{R}}_{\text{Nir}} - {\text{R}}_{\text{Swir}} }}{{{\text{R}}_{\text{Nir}} - {\text{R}}_{\text{Swir}} }}$$

where R_Nir_ and R_Swir_ represent the reflectance of the near infrared bands and short infrared bands, respectively. LSWI has been shown to be sensitive to leaf water content (equivalent water thickness) (Jackson et al. 2004) and soil moisture overtime (Fensholt and Sandholt 2003).

The MODIS 8-day land surface temperature and Emissivity products (MOD11A2) in present works are retrieved at 1 km pixels by the generalized split-window algorithm and at 6 km grids by the day/night algorithm. In the split-window algorithm, emissivity in bands 31 and 32 are estimated from land cover types, atmospheric column water vapor and lower boundary air surface temperature are separated into tractable sub-ranges for optimal retrieval. MODIS LST is a measure of soil or canopy leaf temperature at the surface, which agreed with in situ measured LST within 1 K in the 263–322 K (Wan et al. 2002). Several researches have demonstrated that the satellite-derived LST also has a strong correlation with R_e_ (Rahman et al. 2005; Schubert et al. 2010).

### Carbon flux models

Many previous researches demonstrate that vegetation indices (EVI, LSWI), land surface temperature (LST), photosynthetically active radiation (PAR) are reliable ecological proxies indicating plant phenological stages, canopy stresses (air temperature, soil moisture, vapor pressure deficit) and environmental conditions (incoming solar radiation et al.) in modeling carbon uptake by terrestrial ecosystems. The VI model directly utilizes vegetation indices to estimate carbon flux (*C*_*f*_) in the formation:

3 $$C_{\text{f}} \propto {\text{VI}}$$

Researchers (Gitelson et al. 2006; Sims et al. 2008; Wu et al. 2010) further introduce LST and PAR into model construction on the basis of VI to form the temperature and greenness (TG) and greenness and radiation (GR) models:

4 $$C_{f} \propto {\text{VI}} \times {\text{LST}}$$

5 $$C_{f} \propto {\text{VI}} \times {\text{PAR}}$$

Our research proposes a predictive model incorporating MODIS and ground level data to estimate NEE. The derived MODIS EVI, LSWI, LST and in situ measured PAR are utilized to develop the new model. All the site level data are split into two sets: the training set (2001–2004) that containing 184 points and the test set (2005–2006) that containing 92 points, respectively. The optimum multi-linear regression (MR) model with the maximum determination coefficient (*R*^2^) and the minimal root mean square error (*RMSE*) is subsequently generated after analyzing the relationships between these proxies and NEE and the MR model is shown to substantially have the best performance while comparing to the previous ones.

## Results and discussion

### Correlation and seasonal variation of NEE with PAR, VI and LST

**Fig. 2:**
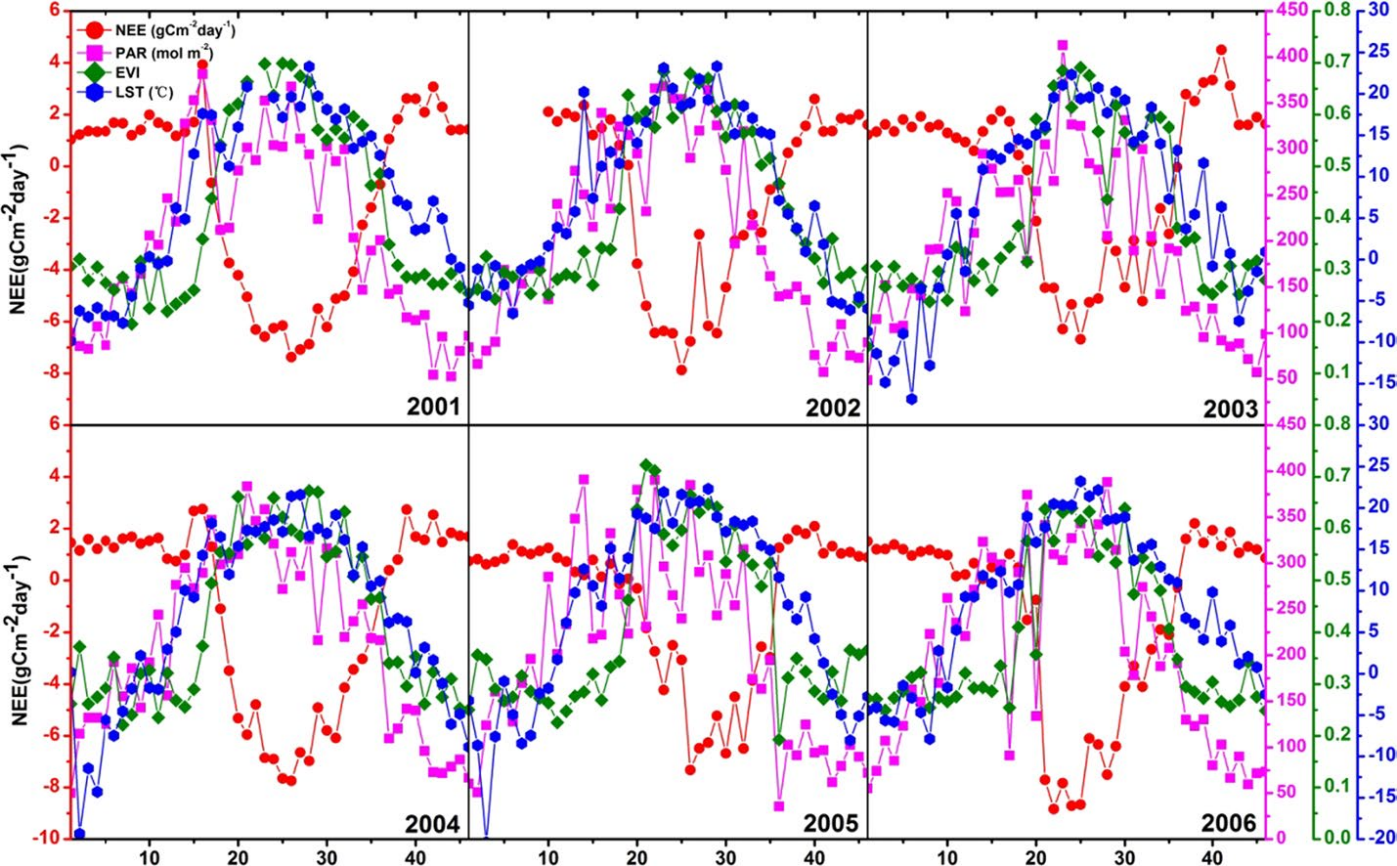
Seasonal dynamics of NEE, PAR, EVI, and LST in 2001–2006 at the Harvard Forest site are shown for each 8 day time interval. The *horizontal axis* represents week of year (WOY) from 1 to 46

Table 1 shows that 2001–2006 time series NEE has significant relationships with all the proxies of PAR, VI (EVI, LSWI) and LST and it has a lower correlation coefficient with LSWI than with EVI. We further examined the seasonal time courses of NEE and the other three variables except LSWI at the Harvard Forest site. Results show that the seasonal fluctuations of NEE can be partly explained by the seasonal variations of photosynthetically active radiation and land surface temperature (Fig. 2). During winter times of 2001–2006 (week of year—WOY ranging from 1 to 16 and from 39 to 46), the photosynthetic activities of the deciduous broadleaf forest were directly inhabited by less incoming sunlight and the low temperature as well as frozen soils because of the shortened sunshine duration (day length) and reduced solar energy received by the ground surface. NEE were above zero and mainly dominated by ecosystem respiration (*R*_*e*_) because GEE were near zero in these months when the canopy is mostly bare after the deciduous leaves (oak and maple) fall off while the remaining few conifers are generally dormant to protect themselves against below freezing temperature. As solar radiation and surface temperature increased from WOY 17 of each year, vegetations started to grow and the intensity of ecosystem photosynthesis activity gradually increased. Subsequently, the photosynthetic carbon fixation exceeded the release of CO_2_ though respiration and NEE reached the maximum peak during WOY 25–30. Later on, NEE declined gradually when forest began to wither accompanied by the reduced photosynthetic radiation and decreased temperature. The strong correlation between NEE and MODIS derived LST may be related with the good relationship between LST and ecosystem respiration but the underlying mechanism remains to be explored.

**Table 1:** Correlations of NEE and ecological proxies PAR, VIs, LST

	NEE	PAR	EVI	LSWI	LST
NEE	1	−0.67*	−0.89*	−0.47*	−0.73*
PAR	−0.67*	1	0.66*	0.17*	0.74*
EVI	−0.89*	0.66*	1	0.43*	0.79*
LSWI	−0.47*	0.17*	0.43*	1	0.06
LST	−0.73*	0.74*	0.79*	0.06	1

2-tailed test of significance is used

* Correlation is significant at the 0.05 level

It could also be seen from Fig. 2 that the MODIS derived EVI had a strong seasonal variation and reflected well the growth status of the deciduous broadleaf forest. EVI successfully captured the beginning and ending of the vegetation growing phase in 2001–2006. It firstly had a significant increase on WOY 15, reached the maximum value during 25–28 and then subsequently gradually declined to remain at a low level after 39. For all phenological indicators, the spring phenology is thought to exert a major effect on carbon balance. Earlier spring onset could consistently although not always significantly lead to higher *R*_*e*_ and GEE for seasonal and annual flux integrals. In response to this, the less increased *R*_*e*_ comparing to GEE would cause an increase NEE of springtime and this phenomenon was more obvious in 2004 than the other years by a magnitude of 2–4 g Cm^−2^ day^−1^.

### Estimating NEE from incorporated MODIS and eddy covariance data

Based on analysis of the training dataset from 2001 to 2004 including PAR, EVI, LSWI and LST for the Harvard Forest we established the traditional VI, TG and GR models as follows:

6 $${\text{NEE}} = 7.351 - 19.605 \times {\text{VI}}$$

7 $${\text{NEE}} = 1.594 - 19.647 \times {\text{VI}} \times {\text{LST}}$$

8 $${\text{NEE}} = 3.291 - 0.043 \times {\text{VI}} \times {\text{PAR}}$$

Here VI refers to EVI because of its better performance than LSWI. It also performs better than all the other single variable models. Then we utilize the step wise linear regression to generate the best performance multi-variable regression model (MR) in which the more significantly correlated variables are incorporated and the less ones are excluded. The derived best performance MR model has with a maximum *R*^2^ of 0.84 and the minimal *RMSE* of 1.33 g Cm^−2^ day^−1^ in the following formation:

9 $${\text{NEE}} = 8.106 - 0.061{\text{PAR}} - 19.037{\text{EVI}} - 0.036{\text{LST}}$$

**Fig. 3:**
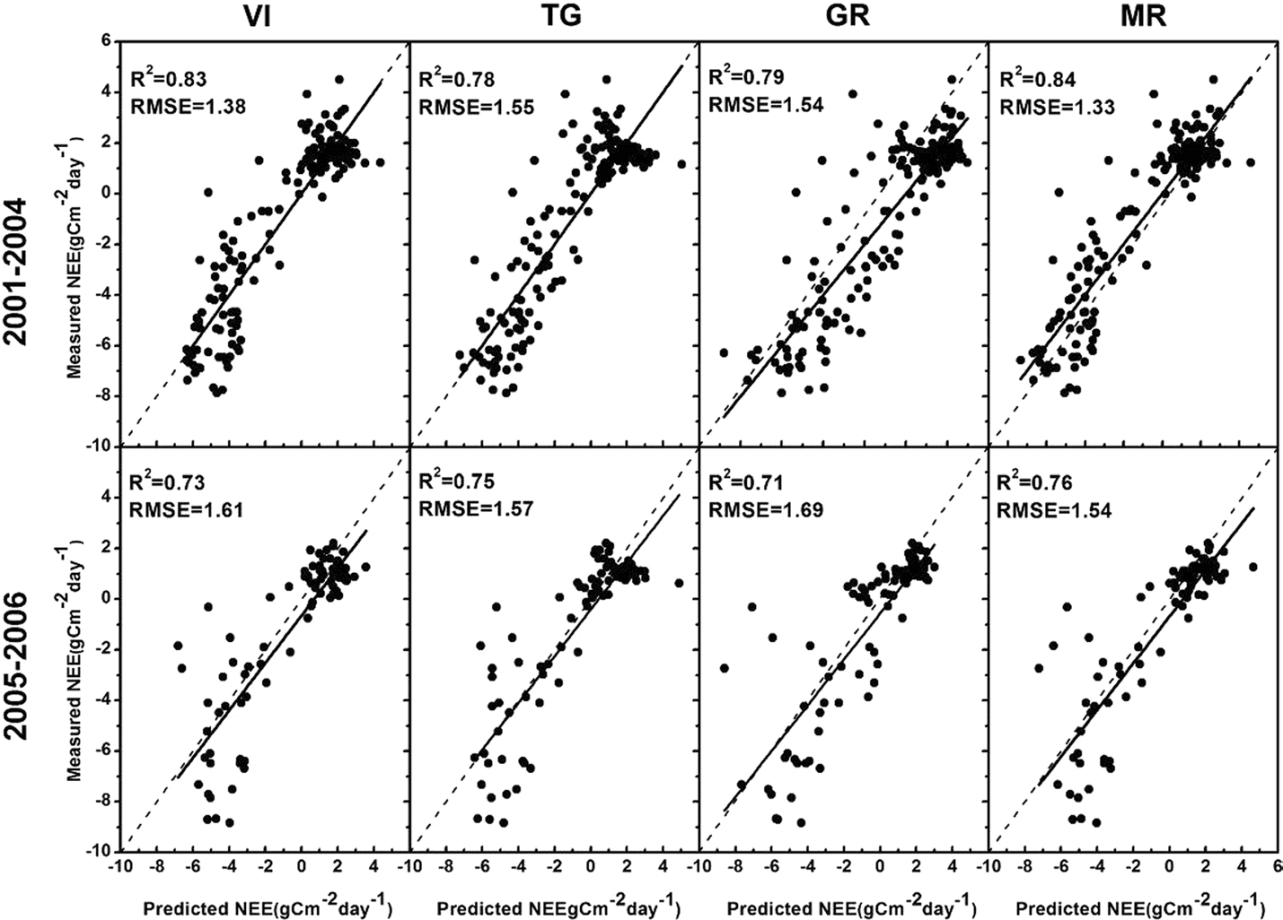
Comparison between the MR model and the other current models for both training (2001–2004) and validation (2005–2006) datasets

The MR model is run at 8-day time scale on the basis of the site-level PAR, EVI, LST to predict NEE and it shows the best performance while comparing the other three models for both training and test datasets (Fig. 3). We further evaluate this model using scatter plots of predicted seasonal NEE versus measured seasonal NEE in 2005–2006.

**Fig. 4:**
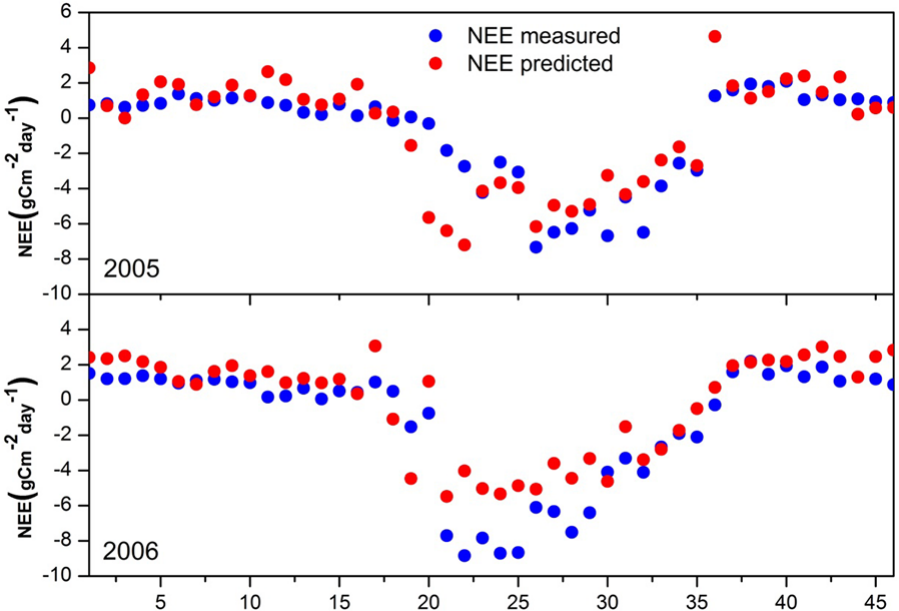
Seasonal variations of MR predicted NEE and tower measured NEE at the Harvard Forest site during the year 2005–2006. The *horizontal axis* represents week of year (WOY) from 1 to 46

The dynamics of seasonal NEE predicted by the MR model based on the integration of MODIS and EC data generally agree well with the measured NEE from the ground CO_2_ tower system in 2005–2006 and this model can substantially improve the estimation accuracy (Figs. 3, 4). It can be seen from Fig. 4 that the residuals are not randomly distributed with low NEE absolute values generally corresponding to lower prediction errors and high NEE absolute values corresponding to higher prediction errors. This phenomenon reveals that the carbon flux measurements uncertainties are directly proportional to the flux magnitudes. There are some discrepancies between the predicted NEE and the corresponding measured values especially during the growing season (WOY 10–35). For example, during WOY 16–32 in 2006, the absolute magnitude of predicted CO_2_ releases into the atmosphere are generally lower than the ground level measurements mainly because that during the growing season especially summer the greenness of canopy leaves and vegetation density reach to high extent under the favorable conditions of sufficient sunlight and precipitation. For the year of 2005 and 2006 at the deciduous dominated Harvard Forest, the predicted results generally agree well with ground observations, therefore, the proposed MR model is shown to be a reliable indicator of seasonal NEE dynamics.

### Uncertainty analysis

Although the proposed model has a better performance than the others in tracking seasonal dynamics of ecosystem carbon exchange for the deciduous-dominated Harvard Forest, it still contains significant uncertainties in NEE estimation.

To match the compositing intervals of MODIS data, weekly NEE data are adopted in our research because seasonal fluctuations of CO_2_ exchange could be well reflected over such a period. However, the magnitudes of carbon sources and sinks fluctuate remarkably on longer timescales such as annual due to geographical location, climate variation, land use change, disturbance by fire and pests, and age distribution as well as species composition of the ecosystem (Johnson et al. 2007). Therefore, the reliability of the MR model needs to be further validated across multiple biomes and over various time scales.

The proxies we selected here to estimate NEE in this study only includes PAR, EVI, and LST, it provides the opportunity to assess CO_2_ exchange directly from remote sensing observations since MODIS already produces EVI and LST products and remote estimation of PAR from MODIS aerosol type and atmospheric conditions would further make the model attractive for operational applications based on entirely remote sensing data (Liang et al. 2006). However, other factors that also have significant influences on CO_2_ exchange are not fully evaluated. NEE is the sum of canopy photosynthesis (GEE) and ecosystem respiration (*R*_*e*_), and GEE is related with sunlight, temperature, ambient humidity, canopy growth and nutrient status while *R*_*e*_ has been identified to have connections with soil moisture, air temperature, nutrient availability, stocks of living and dead biomass, seasonal carbon allocation as well as ecosystem productivity (Boone et al. 1998). Therefore, further research may focus on the underlying mechanism of ecophysiological interactions between the ecosystem and the environment variables.

## Conclusion

Accurate estimation of terrestrial carbon exchange is of great importance for regional and global studies of carbon balance. We compare a multiple-linear regression model which relates vegetation indices and several environmental factors with the site-level NEE representing carbon flux exchange, with other models proposed in the literature in this mixed temperate forest. Results show that MR model could track seasonal fluctuations of ecosystem carbon exchange better than the others at the deciduous-dominated Harvard Forest site level. The discrepancies between model simulated NEE and measured NEE may be attributed with different dynamical ranges in EVI and the relatively importance of various environmental factors. Given the best performance in the accuracy of carbon flux exchange estimation by the model developed here, it is worthwhile to evaluate the efficacy of this method across different ecosystems at various time intervals for scaling up plot-level NEE of CO_2_ to large spatial areas.
